# Axl and autophagy LC3 expression in tumors is strongly associated with clinical prognosis of hepatocellular carcinoma patients after curative resection

**DOI:** 10.1002/cam4.2229

**Published:** 2019-05-16

**Authors:** Chia‐Chang Hsu, Pei‐Min Hsieh, Yaw‐Sen Chen, Gin‐Ho Lo, Hung‐Yu Lin, Chia‐Yen Dai, Jee‐Fu Huang, Wan‐Long Chuang, Yao‐Li Chen, Ming‐Lung Yu, Chih‐Wen Lin

**Affiliations:** ^1^ School of Medicine, College of Medicine I‐Shou University Kaohsiung Taiwan; ^2^ Health Examination Center, E‐Da Hospital I‐Shou University Kaohsiung Taiwan; ^3^ Division of Gastroenterology and Hepatology, E‐Da Dachang Hospital I‐Shou University Kaohsiung Taiwan; ^4^ Department of Surgery, E‐Da Hospital I‐Shou University Kaohsiung Taiwan; ^5^ Division of Gastroenterology and Hepatology, Department of Medicine E‐Da Hospital, I‐Shou University Kaohsiung Taiwan; ^6^ Center for Infectious Disease and Cancer Research Kaohsiung Medical University Kaohsiung Taiwan; ^7^ Graduate Institute of Medicine, College of Medicine Kaohsiung Medical University Kaohsiung Taiwan; ^8^ Hepatobiliary Division, Department of Internal Medicine, and Hepatitis Center Kaohsiung Medical University Hospital, Kaohsiung Medical University Kaohsiung Taiwan; ^9^ Division of General Surgery, Department of Surgery Changhua Christian Hospital Changhua Taiwan; ^10^ Institute of Biomedical Sciences National Sun Yat‐Sen University Kaohsiung Taiwan; ^11^ Center for Intelligent Drug Systems and Smart Bio‐devices, College of Biological Science and Technology National Chiao Tung University Hsin‐Chu Taiwan; ^12^ School of Chinese Medicine, College of Chinese Medicine China Medical University Taichung Taiwan; ^13^ Research Center for Traditional Chinese Medicine China Medical University Hospital Taichung Taiwan

**Keywords:** autophagy LC3, Axl, hepatocellular carcinoma, overall survival, predictors, recurrence

## Abstract

**Background:**

The role of Axl and LC3 as predictors of tumor recurrence and overall survival (OS) after hepatocellular carcinoma (HCC) resection remains unclear.

**Methods:**

We retrospectively included 535 HCC patients who underwent hepatectomy from 2010 to 2014 in this study. Axl and the autophagy‐related marker LC3 were immunohistochemically assessed in tumors.

**Results:**

Axl expression was significantly associated with advanced clinicopathological features, including cirrhosis, microvascular invasion, macrovascular invasion, tumor size, BCLC stage, recurrence, and mortality. HCC recurrence occurred in 245 patients, and 219 patients died. The 5‐year cumulative incidences of HCC recurrence and OS rate after HCC resection were 53.3% and 58.8%, respectively. In the Cox proportional analyses, high Axl expression and high LC3 expression were significantly associated with HCC recurrence (hazard ratio [HR]: 3.85, 95% confidence interval [CI]: 2.95‐5.02, *P* < 0.001; and HR: 0.38, 95% CI: 0.26‐0.55, *P* < 0.001, respectively). In addition, HCC recurrence (HR: 2.87, 95% CI: 2.01‐4.01, *P* < 0.0001), microvascular invasion (HR: 1.85, 95% CI: 1.08‐3.19, *P* = 0.026), hepatitis B virus‐related HCC (HR: 1.77, 95% CI: 1. 21‐2.56, *P* = 0.003), high Axl expression (HR: 1.66, 95% CI: 1.41‐1.97, *P* < 0.0001), antiviral therapy (HR: 0.54, CI: 0.38‐0.76, *P* < 0.001) and LC3 expression (HR: 0.41, 95% CI: 0.28‐0.58, *P* < 0.001) were significantly associated with mortality. Furthermore, patients with a combination of high Axl and low LC3 expression had the highest risk of HCC recurrence (HR: 6.53, 95% CI: 4.11‐10.4, *P* < 0.001) and mortality (HR: 6.66, 95% CI: 4.07‐10.9, *P* < 0.001). In patients with high Axl, low LC3, and combined high Axl and low LC3 expression, the 5‐year cumulative incidences of HCC recurrence and OS rate were 77.9%, 73.3%, and 90.0% and 28.8%, 26.7%, and 16.8%, respectively.

**Conclusion:**

High Axl expression in tumors is associated with aggressive tumor behavior and worse clinical outcomes. Furthermore, the combination of high Axl and low LC3 expression significantly predicts poorer prognosis for HCC patients who underwent hepatectomy.

## 1 INTRODUCTION

Hepatocellular carcinoma (HCC) is the third common cause of cancer‐related death in the world.^1^, ^2^ In Taiwan, viral‐ and alcohol‐related cirrhosis frequently result in HCC.^3^ Moreover, HCC is hard to diagnose at the very early and early stages, resulting in higher mortality rates worldwide.^1^, ^2^ Although the 5‐year survival rate of individuals diagnosed in the early stage exceeded 50% after curative resection, these patients still had a high recurrence rate of approximately 60% after curative resection.^4^, ^5^, ^6^, ^7^, ^8^ The highly sensitive marker alpha‐fetoprotein (AFP) predicts clinical outcome in HCC patients after hepatectomy, but the result is still unsatisfactory.^9^ Hence, the identification of biomarkers for HCC recurrence and overall survival (OS) could help improve the clinical prognosis of HCC patients undergoing hepatectomy.

Axl, a member of the Tyro3, Mer, and Axl family of tyrosine kinase receptors, regulates some aspects of cancer biology.^10^ The Axl‐mediated signaling pathway is frequently affected in the development and progression of various cancers, including brain cancer, breast cancer, and pancreatic cancer.^11^, ^12^, ^13^ Recently, Reichl et al reported that high serum levels of soluble Axl are correlated with vascular involvement and lymph node metastasis, and the serum level of soluble Axl is a potential biomarker for the early diagnosis of HCC and the early prediction of HCC recurrence.^14^ Moreover, Wu et al demonstrated that Axl overexpression/hyperactivation plays a major role in epithelial‐to‐mesenchymal transition, cancer chemotherapy resistance, and increased metastasis, all of which implicate Axl as an important target.^15^ Several studies showed that Axl may be a negative predictor for HCC patients, and high Axl expression was positively associated with differentiation, lymph node metastasis, higher recurrence rates, and lower survival rates in HCC patients.^16^, ^17^ Furthermore, Axl expression was associated with increased tumor invasion and predicted a worse prognosis for HCC patients undergoing resection.^18^ Our previous studies showed that high LC3 expression in the liver and tumor microenvironments is strongly correlated with higher OS and lower HCC recurrence.^7^, ^8^ However, whether Axl expression is associated with clinical prognosis in HCC patients remains largely unknown. In addition, the role of Axl and the autophagy‐related marker LC3 in OS and HCC recurrence is not clear. Hence, we investigate the impact of Axl and LC3 expression on tumor recurrence and OS in a large cohort of HCC patients who underwent curative resection.

## 2 MATERIALS AND METHODS

### 2.1 Patients and follow‐up

We retrospectively collected the data from 535 HCC patients who underwent resection from 2010 to 2014 at Changhua Christian Hospital, Changhua, or E‐Da Hospital, I‐Shou University, Kaohsiung, Taiwan. All participants were regularly followed‐up every three to six months after surgery. The last recorded follow‐up was on 31 December 2016. OS was the duration from the date of hepatectomy to the date of death or the last visit. Survival data were censored on the end‐data of follow‐up. Time to recurrence was the duration from the date of hepatectomy to the date of recurrence. HCC recurrence was based on histology or at least two typical HCC imaging methods according to the HCC guidelines of the American Association for the Study of Liver Disease.^19^

The clinicopathological features of the patients, such as demographic data and tumor characteristics, were recorded. Antiviral treatment was defined as patients with hepatitis B virus (HBV), hepatitis C virus (HCV), or dual HBV/HCV infection who received therapy with oral nucleosides, pegylated interferon‐based therapy or direct‐acting agents according to the guidelines of the Taiwan Association for the Study of the Liver. This research was approved by the Institutional Review Boards of Changhua Christian Hospital (091107) and E‐DA Hospital (EMRP38104N). The research was conducted according to the guidelines of the International Conference on Harmonization for Good Clinical Practice. All participants were adults and provided written informed consent for study participation.

### 2.2 Immunohistochemical staining and scoring

Tissue microarrays were constructed as previously described^7^ and subjected to immunohistochemistry (IHC). The tissues were stained with an anti‐Axl antibody (Santa Cruz, CA) and an anti‐LC3 antibody (Novus Biologicals, CO). Axl and LC3 expression was quantitated using the semiquantitative immunoreactive scoring system (IRS) as described previously,^20^ and the expression was defined as either negative (IRS < 2) or positive (IRS ≥ 2) according to the percentage and intensity scores. All tissues were independently assessed by two investigators who were blinded to the clinical data.

### 2.3 Data analysis and statistics

Numerical data are presented as the mean (range). Categorical data are described using numbers (percentages). Continuous variables were categorized based on the mean values or the limits of normal ranges in Cox regression models. Fisher's exact test was used for comparison of continuous data. Pearson's χ^2^ test was used for comparison between categorical variables. Tumor recurrence and OS were evaluated by Kaplan‐Meier analyses and Cox proportional hazards regression analyses. A *P* < 0.05 indicated statistical significance. All statistical analyses were performed with Statistics Package for Social Science (SPSS) software (version 23.0, SPSS Inc, Chicago, IL).

## 3 RESULTS

### 3.1 Baseline demographic data

The demographic and clinicopathological features of the 535 patients are presented in Table 1. First, 46.7% of the HCC patients had HBV, and 28.4% had HCV. One‐third of the patients had liver cirrhosis. Regarding tumor stage, 36.1% and 16.4% of the patients were Barcelona clinic liver cancer (BCLC) stage B‐C and TNM stage III‐IV, respectively. In addition, 91.6% of the HCC tissues showed high LC3 expression.

**Table 1:** Basic demographic data of all patients and correlations between tumor Axl expression and clinicopathologic characteristics

Characteristics	All patients (n = 535)	Axl‐low (n = 305)	Axl‐high (n = 230)	*P*‐value^a^
Gender				
Female	144 (26.9)	70 (23.0)	74 (32.2)	0.017
Male	391 (73.1)	235 (77.0)	156 (67.8)	
Age (y)	63.1 ± 11.5	62.9 ± 12.1	63.4 ± 10.6	0.580
HCC etiology				
HCV	152 (28.4)	98 (32.1)	54 (23.5)	0.010
HBV	250 (46.7)	143 (46.9)	107 (46.5)	
Non‐HBV/HCV	112 (20.9)	50 (16.4)	62 (27.0)	
HBV + HCV	21 (3.9)	14 (4.6)	7 (3.0)	
AFP (ng/dL)	2797 ± 13215	3019 ± 13076	2504 ± 13418	0.410
Liver cirrhosis				
Negative	362 (67.7)	194 (63.6)	168 (73.0)	0.021
Positive	173 (32.3)	111 (36.4)	62 (27.0)	
Antiviral therapy				
Negative	185 (43.7)	103 (40.4)	82 (48.8)	0.088
Positive	238 (56.3)	152 (59.6)	86 (51.2)	
Edmondson‐steiner grade				
I‐II	51 (9.5)	30 (9.8)	21 (9.1)	0.783
III‐IV	484 (90.5)	275 (90.2)	209 (90.9)	
Macrovascular invasion				
Negative	424 (79.3)	258 (84.6)	166 (72.2)	<0.001
Positive	111 (20.7)	47 (15.4)	64 (27.8)	
Microvascular invasion				
Negative	289 (54.0)	186 (61.0)	103 (44.8)	<0.001
Positive	246 (46.0)	119 (39.0)	127 (55.2)	
Tumor count				
One	438 (81.9)	247 (81.0)	191 (83.0)	0.540
Multiple	97 (18.1)	58 (19.0)	39 (17.0)	
Tumor size				
<5 cm	352 (65.8)	216 (70.8)	136 (59.1)	0.005
≥5 cm	183 (34.2)	89 (29.2)	94 (40.9)	
TNM stage				
I‐II	447 (83.6)	261 (85.6)	186 (80.9)	0.146
III‐IV	88 (16.4)	44 (14.4)	44 (19.1)	
BCLC stage				
0‐A	342 (63.9)	213 (62.3)	129 (37.7)	0.001
B‐C	193 (36.1)	92 (47.7)	88 (52.3)	
Recurrence				
No	290 (54.2)	203 (66.6)	87 (37.8)	<0.001
Yes	245 (45.8)	102 (33.4)	143 (62.2)	
Death				
No	316 (59.1)	232 (76.1)	84 (36.5)	<0.001
Yes	219 (40.9)	73 (23.9)	146 (63.5)	
LC3 in tumors				
Low	45 (8.4)	16 (5.2)	29 (12.6)	0.002
High	490 (91.6)	289 (94.8)	201 (87.4)	

Data are shown as the mean ± standard deviation or number (%).

Abbreviations: AFP, alpha‐fetoprotein; BCLC, Barcelona clinic liver cancer; HBV, Hepatitis B virus; HCV, hepatitis C virus.

a *P*‐value for the comparison between the Axl‐low and Axl‐high groups.

### 3.2 Axl expression is associated with advanced clinicopathological features

Within the cohort, 43.0% (230) and 57.0% (305) of the 535 HCC tissues had high and low Axl expression, respectively, as presented in Table 1. Axl expression was significantly correlated with advanced clinicopathological features, including cirrhosis (111 [36.4%] vs 62 [27.0%], *P* = 0.021), microvascular invasion (119 [39.0%] vs 127 [55.2%], *P* < 0.001), macrovascular invasion (47 [15.4%] vs 64 [27.8%], *P* < 0.001), tumor size ≥ 5 cm (89 [29.2%] vs 94 [40.9%], *P* = 0.005), BCLC stage B/C (92 [47.7%] vs 88 [52.3%], *P* = 0.001), recurrence (102 [33.4%] vs 143 [62.2%], *P* < 0.001), and death (73 [23.9%] vs 146 [63.5%], *P* < 0.001).

### 3.3 Predictive factors associated with tumor recurrence in HCC patients who underwent hepatectomy

HCC recurrence occurred in 245 patients, and the HCC recurrence rate at 1, 3, 5, and 7 years after hepatectomy was 9.7%, 33.9%, 53.3% and 66.3%, respectively. In the univariate analysis, macrovascular invasion, liver cirrhosis, high Axl expression in tumors and low LC3 expression in tumors were significantly correlated with increased HCC recurrence (Table 2).

**Table 2:** Univariate and multivariate analyses of factors associated with tumor recurrence

Characteristics	Univariate analyses			Multivariate analyses	
	Without recurrence (n = 290)	With recurrence (n = 245)	*P*‐value	HR (95% CI)	*P*‐value
Gender					
Female	69 (23.8)	75 (30.6)	0.076		
Male	221 (76.2)	170 (69.4)			
Age (y)					
<65	104 (35.9)	68 (27.8)	0.056		
≥65	186 (64.1)	177 (72.2)			
HCC etiology					
HCV	85 (29.3)	67 (27.3)	0.826		
HBV	132 (45.5)	118 (48.2)			
Non‐HBV/HCV	60 (20.7)	52 (21.2)			
HBV + HCV	13 (4.5)	8 (3.3)			
AFP (ng/dL)					
<400	236 (81.4)	202 (82.4)	0.749		
≥400	54 (18.6)	43 (17.6)			
Liver cirrhosis					
Negative	211 (72.8)	151 (61.6)	0.006	1	0.306
Positive	79 (27.2)	94 (38.4)		0.86 (0.65‐1.14)	
Antiviral therapy					
Negative	92 (40.0)	93 (48.2)	0.091		
Positive	138 (60.0)	100 (51.8)			
Edmondson‐steiner grade					
I‐II	34 (11.7)	17 (6.9)	0.060		
III‐IV	256 (88.3)	228 (93.1)			
Macrovascular invasion					
Negative	240 (82.8)	184 (75.1)	0.030	1	0.192
Positive	50 (17.2)	61 (24.9)		1.21 (0.91‐1.64)	
Microvascular invasion					
Negative	158 (54.5)	131 (53.5)	0.815		
Positive	132 (45.5)	114 (46.5)			
Tumor count					
One	238 (82.1)	200 (81.6)	0.896		
Multiple	52 (17.9)	45 (18.4)			
Tumor size					
<5 cm	191 (65.9)	161 (65.7)	0.971		
≥5 cm	99 (34.1)	84 (34.3)			
TNM stage					
I‐II	241 (83.1)	206 (84.1)	0.761		
III‐IV	49 (16.9)	39 (15.9)			
BCLC stage					
0‐A	185 (63.8)	157 (64.1)	0.945		
B‐C	105 (36.2)	88 (35.9)			
Axl in tumors					
Low	203 (70.0)	102 (41.6)	<0.001	1	
High	87 (30.0)	143 (58.4)		3.85 (2.95‐5.02)	<0.001
LC3 in tumors					
Low	11 (3.8)	34 (13.9)	<0.001	1	<0.001
High	279 (96.2)	211 (86.1)		0.38 (0.26‐0.55)	

Data are shown as the mean ± standard deviation or number (%).

Abbreviations: AFP, alpha‐fetoprotein; BCLC, Barcelona clinic liver cancer; CI, confidence interval; HBV, hepatitis B virus; HCV, hepatitis C virus; HR, hazard ratio.

The multivariate Cox regression analysis revealed that high Axl expression in tumors was significantly correlated with increased HCC recurrence (hazard ratio [HR]: 3.85, 95% confidence interval [CI]: 2.95‐5.02, *P* < 0.001), but high LC3 expression in tumors was significantly correlated with decreased HCC recurrence (HR: 0.38, 95% CI: 0.26‐0.55, *P* < 0.001), as shown in Table 2.

**Figure 1:**
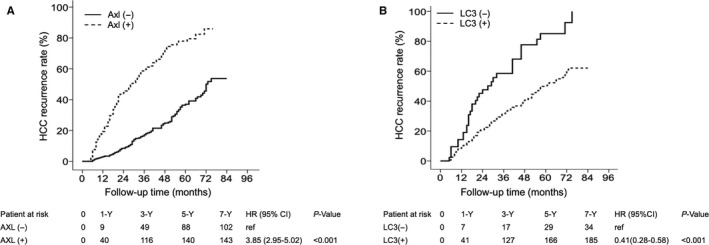
Cumulative incidence of tumor recurrence according to Axl and LC3 expression in tumors. The cumulative incidence of HCC recurrence is significantly lower in patients with low Axl expression than in those with high Axl expression (A). The cumulative incidence of HCC recurrence is significantly lower in patients with high LC3 expression than in those with low LC3 expression (B). −, low; +, high; HCC, hepatocellular carcinoma.

Patients with high Axl expression in tumors had a significantly higher HCC recurrence rate than those with low Axl expression in tumors by Kaplan‐Meier analysis. In patients with high and low Axl expression in tumors, the 1‐, 3‐, 5‐ and 7‐year HCC recurrence rates were 19.3%, 58.6%, 77.9% and 85.9% and 3.0%, 17.2%, 37.1% and 53.7%, respectively (Figure 1A). In addition, patients with low LC3 expression in tumors had a significantly higher HCC recurrence rate than those with high LC3 expression in tumors. In patients with low and high LC3 expression in tumors, the 1‐, 3‐, 5‐ and 7‐year HCC recurrence rates were 15.6%, 39.2%, 73.3% and 94.9% and 8.4%, 26.7%, 37.9% and 69.5%, respectively (Figure 1B).

### 3.4 Predictive factors associated with OS in HCC patients who underwent hepatectomy

The median follow‐up time was 42 months, and 219 patients died. The OS rates at 1, 3, 5 and 7 years after resection were 91.0%, 72.3%, 58.8%, and 27.7%, respectively. In the univariate analysis, the following factors were significantly correlated with OS: sex, HBV‐related HCC, cirrhosis, antiviral treatment, the presence of macrovascular and microvascular invasion, primary tumor size, BCLC stage, HCC recurrence, Axl expression in tumors and LC3 expression in tumors.

The multivariate Cox regression analysis showed that patients with HCC recurrence had the highest mortality (HR: 2.87, 95% CI: 2.01‐4.01, *P* < 0.001), followed by those with microvascular invasion (HR: 1.85, 95% CI: 1.08‐3.19, *P* = 0.026), HBV‐related HCC (HR: 1.77, 95% CI: 1.21‐2.56, *P* = 0.003), and high Axl expression in tumors (HR: 1.66, 95% CI: 1.41‐1.97, *P* < 0.0001); these data are summarized in Table 3. Mortality was also significantly decreased in patients receiving antiviral treatment (HR: 0.54, CI: 0.38‐0.76, *P* < 0.001) and in those with high LC3 expression in tumors (HR: 0.41, 95% CI: 0.28‐0.58, *P* < 0.001), as shown in Table 3.

**Table 3:** Univariate and multivariate analyses of factors associated with mortality of hepatocellular carcinoma patients who underwent curative resection

Characteristics	Univariate analyses			Multivariate analyses	
	No mortality (n = 316)	Mortality (n = 219)	*P*‐value	HR (95% CI)	*P*‐value
Gender					
Female	74 (23.4)	70 (32.0)	0.030	1	0.143
Male	242 (76.6)	149 (68.0)		0.78 (0.55‐1.09)	
Age (y)					
<65	107 (33.9)	65 (29.7)	0.309		
≥65	209 (66.1)	154 (70.3)			
HCC etiology					
HCV	102 (32.3)	50 (22.8)	0.015	1	
HBV	141 (44.6)	109 (49.8)		1.77 (1.21‐2.56)	0.003
Non‐HBV/HCV	57 (18.0)	55 (25.1)		0.99 (0.38‐2.58)	0.990
HBV + HCV	16 (5.1)	5 (2.3)		0.92 (0.62‐1.23)	0.753
AFP (ng/dL)					
<400	260 (82.3)	178 (81.3)	0.768		
≥400	56 (17.7)	41 (18.7)			
Liver cirrhosis					
Negative	227 (71.8)	135 (61.6)	0.013	1	0.316
Positive	89 (28.2)	84 (38.4)		1.20 (0.84‐1.69)	
Antiviral therapy					
Negative	98 (37.8)	87 (53.0)	0.002	1	<0.001
Positive	161 (62.2)	77 (47.0)		0.54 (0.38‐0.76)	
Edmondson‐steiner grade					
I‐II	28 (8.9)	23 (10.5)	0.551		
III‐IV	288 (91.1)	196 (89.5)			
Macrovascular invasion					
Negative	261 (82.6)	163 (74.4)	0.022	1	0.528
Positive	55 (17.4)	56 (25.6)		0.85 (0.52‐1.40)	
Microvascular invasion					
Negative	185 (58.5)	104 (47.5)	0.012	1	0.026
Positive	131 (41.5)	115 (52.5)		1.85 (1.08‐3.19)	
Tumor count					
One	250 (79.1)	188 (85.8)	0.052		
Multiple	66 (20.9)	31 (14.2)			
Tumor size					
<5 cm	220 (69.6)	132 (60.3)	0.026	1	0.475
≥5 cm	96 (30.4)	87 (39.7)		1.21 (0.72‐2.01)	
TNM stage					
I‐II	262 (82.9)	185 (84.5)	0.722		
III‐IV	54 (17.1)	34 (15.5)			
BCLC stage					
0‐A	214 (67.7)	128 (58.4)	0.035	1	0.613
B‐C	102 (32.3)	91 (41.6)		0.86 (0.47‐1.56)	
HCC recurrence status					
Negative	226 (71.5)	64 (29.2)	<0.0001	1	<0.001
Positive	90 (28.5)	155 (70.8)		2.87 (2.01‐4.10)	
Axl in tumors					
Low	232 (73.4)	73 (33.3)	<0.001	1	<0.001
High	84 (26.6)	146 (66.7)		1.66 (1.41‐1.97)	
LC3 in tumors					
Low	11 (3.5)	34 (15.5)	<0.001	1	<0.001
High	305 (96.5)	185 (84.5)		0.41 (0.28‐0.58)	

Data are shown as the mean ± standard deviation or number (%).

Abbreviations: AFP, alpha‐fetoprotein; BCLC, Barcelona clinic liver cancer; CI, confidence interval; HBV, hepatitis B virus; HCV, hepatitis C virus; HR, hazard ratio.

**Figure 2:**
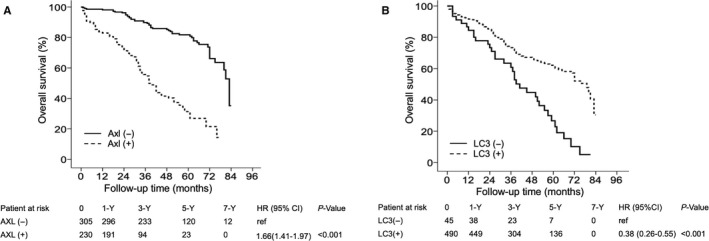
Cumulative incidence of overall survival according to Axl and LC3 expression in tumors. The cumulative incidence of overall survival is significantly higher in patients with low Axl expression than in those with high Axl expression (A). The cumulative incidence of overall survival is significantly higher in patients with high LC3 expression than in those with low LC3 expression (B). −, low; +, high; HCC, hepatocellular carcinoma.

Patients with high Axl expression in tumors had significantly lower OS than those with low Axl expression in tumors. In patients with high and low Axl expression in tumors, the 1‐, 3‐, 5‐ and 7‐year OS rates were 83.0%, 51.3%, 28.8% and 15.6% and 97.0%, 87.8%, 79.3% and 38.4%, respectively (Figure 2A). In addition, patients with low LC3 expression in tumors had significantly lower OS than those with high LC3 expression in tumors. In patients with low and high LC3 expression in tumors, the 1‐, 3‐, 5‐ and 7‐year OS rates were 84.4%, 60.8%, 26.7% and 5.1% and 91.6%, 73.3%, 62.1% and 30.5%, respectively (Figure 2B).

### 3.5 Tumor recurrence and OS according to combined Axl and LC3 expression

**Figure 3:**
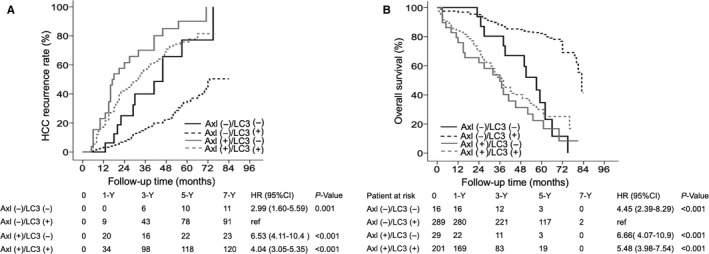
Cumulative incidence of tumor recurrence and overall survival according to the combination of Axl and LC3 expression in tumors. Patients with +/+, −/− or +/+ Axl/LC3 expression had a significantly higher incidence of recurrence than patients with −/+ Axl/LC3 expression (A). Patients with +/−, −/− or +/+ Axl/LC3 expression had significantly lower overall survival than patients with −/+ Axl/LC3 expression (B). −, low; +: high; CI, confidence interval; HCC, hepatocellular carcinoma, HR, hazard ratio.

Next, the combination of Axl and LC3 expression in tumors was analyzed. For HCC recurrence, compared to patients (n = 289) with low Axl and high LC3 expression in tumors, patients (n = 29) with high Axl and low LC3 expression had the highest risk of HCC recurrence (HR: 6.53, 95% CI: 4.11‐10.4, *P* < 0.001), followed by those (n = 201) with high Axl and high LC3 expression (HR: 4.04, 95% CI: 3.05‐5.35, *P* < 0.001) and those (n = 16) with low Axl and low LC3 expression (HR: 2.99, 95% CI: 1.60‐5.59, *P* = 0.001). Patients with low Axl and high LC3 expression in tumors had 1‐, 3‐, 5‐ and 7‐year HCC recurrence rates of 3.2%, 15.9%, 34.9% and 50.4%, respectively. Compared with this group, the low Axl/low LC3 group (0%, 40.0%, 77.1% and 100%, respectively), high Axl/high LC3 group (18.8%, 56.9%, 75.7% and 81.5%, respectively) and high Axl/low LC3 group (23.1%, 70.1%, 90.0% and 100%, respectively) had significantly higher HCC recurrence rates (Figure 3A).

Moreover, compared with patients with low Axl and high LC3 expression in tumors, patients with high Axl and low LC3 expression had the highest risk of mortality (HR: 6.66, 95% CI: 4.07‐10.9, *P* < 0.001), followed by those with high Axl and high LC3 expression (HR: 5.48, 95% CI: 3.98‐7.54, *P* < 0.001) and those with low Axl and low LC3 expression (HR: 4.45, 95% CI: 2.39‐8.29, *P* < 0.001).

The results showed that patients with low Axl and high LC3 expression in HCC tissues had 1‐, 3‐, 5‐ and 7‐year OS rates of 96.9%, 88.2%, 82.2% and 41.6%, respectively. Compared with this group, the low Axl/low LC3 group (100%, 80.4%, 34.7% and 0%, respectively), high Axl/high LC3 group (84.1%, 51.7%, 30.0% and 16.8%, respectively) and high Axl/low LC3 group (75.9%, 49.2%, 16.8% and 8.4%, respectively) had significantly lower OS rates (Figure 3B).

## 4 DISCUSSION

In this study, 535 HCC patients who underwent curative resection were analyzed to identify predictive factors for HCC recurrence and OS. High Axl expression in tumors was significantly associated with advanced clinicopathological features, high HCC recurrence rates, and low OS rates. In addition, low LC3 expression in tumors was also significantly correlated with high HCC recurrence and low OS rates. Furthermore, the combined expression of Axl and LC3 in tumors was analyzed. Patients with high Axl and low LC3 expression in tumors had the highest HCC recurrence (HR: 6.53; *P* < 0.001) and mortality rates (HR: 6.66; *P* < 0.001). These findings suggest that Axl and LC3 expression levels in tumors may serve as predictors of HCC recurrence and OS after hepatectomy for HCC.

Several studies showed that high Axl expression in HCC patients was positively associated with more aggressive tumor invasiveness, a high risk of tumor recurrence, lymph node metastasis, and lower survival.^16^, ^17^, ^18^ Our study demonstrated that Axl expression was significantly correlated with advanced clinicopathological features, including cirrhosis, microvascular invasion, macrovascular invasion, tumor size, BCLC stage, recurrence, and mortality in the univariate analysis. In the multivariate analysis, Axl expression was significantly correlated with tumor recurrence and mortality (data not shown). In addition, high Axl expression in tumors was strongly correlated with high HCC recurrence and low OS rates. The 5‐year cumulative incidences of HCC recurrence and OS were 77.9% and 28.8%, respectively, in patients with high Axl expression. Our study results are consistent with those of previous reports.^16^, ^17^, ^18^ High Axl expression in tumors corresponded to more aggressive tumor behavior and a worse clinical prognosis. Axl expression in tumors may be a negative predictor of HCC recurrence and OS and may predict a worse clinical prognosis in HCC patients.

Previous studies of LC3 expression in terms of clinical outcomes have reported controversial results.^21^, ^22^, ^23^ Our previous work showed that high LC3 levels in both the liver and tumor microenvironments are significantly correlated with decreased HCC recurrence and increased OS after surgical resection. High LC3 expression seems to have a protective effect on clinical outcomes, including HCC recurrence and OS after hepatectomy.^7^, ^8^ Our present study demonstrated that low LC3 expression in tumors is correlated with high HCC recurrence and low OS rates. The 5‐year cumulative incidences of HCC recurrence and OS were 73.3% and 26.7%, respectively, in patients with low LC3 expression. LC3 expression in tumors can predict the clinical outcomes of HCC patients after hepatectomy and may exert a protective effect against HCC recurrence, thus improving OS.

The impact of Axl and LC3 expression in tumors on HCC recurrence and OS has never been reported in the literature. Hence, we revealed that the combination of high Axl expression and low LC3 expression in tumors had additional deleterious effects on HCC recurrence and OS. Our results show the important role of Axl and LC3 expression in tumors in the prognosis of HCC recurrence and OS. The study is the first to demonstrate that Axl and LC3 expression in tumors is significantly associated with HCC recurrence and OS. The combination of high Axl and low LC3 expression in tumors significantly increases the risk of HCC recurrence and mortality in HCC patients after hepatectomy.

Our study showed that high Axl expression in tumors increased HCC recurrence (HR: 3.85) and mortality (HR: 1.66) compared with low Axl expression and that high LC3 expression in tumors decreased HCC recurrence (HR: 0.38) and mortality (HR: 0.41) compared with low LC3 expression. Furthermore, high Axl and low LC3 expression in tumors increased HCC recurrence (HR: 6.53) and mortality (HR: 6.66) compared with low Axl and high LC3 expression. We aimed to find better markers to precisely predict HCC recurrence and mortality. Axl expression in tumors was a better predictor than LC3 expression of HCC recurrence. The combination of Axl and LC3 expression in tumors by IHC staining significantly predicted HCC recurrence and mortality compared with the expression of either marker alone. The combination of two markers was a better predictor of HCC recurrence and mortality than a single marker. In clinical practice, tumor tissues may be subjected to IHC staining for Axl and LC3 after hepatectomy. Patients with high Axl and low LC3 expression in tumors will be identified as having a higher risk of HCC recurrence and mortality than those with low Axl and high LC3 expression in tumors. Close postoperative follow‐up is suggested for this patient population to increase the detection rate of early‐stage HCC, and further aggressive management may prolong the survival of these high‐risk patients.

This study had some limitations. First, this study was retrospective, which could have introduced unintended bias. Second, cirrhotic patients composed only one‐third of our cohort, while other studies have included a majority of cirrhosis patients (80%). This difference in prevalence of cirrhosis may have affected the generalizability of the outcomes. Finally, the underlying mechanism of Axl and LC3 expression related to carcinogenesis and clinical prognosis needs to be further investigated in vivo and in vitro.

In summary, high Axl expression in tumors is significantly associated with advanced clinicopathological features, high HCC recurrence rate, and low OS rate. In addition, low LC3 expression in tumors is also significantly associated with high HCC recurrence and low OS rates. Furthermore, patients with a combination of high Axl expression and low LC3 expression in tumors had higher HCC recurrence and lower OS rates. This study is the first to demonstrate that the combined Axl and LC3 expression pattern in the tumor microenvironment is critical for predicting HCC recurrence and OS. Analysis of Axl and LC3 expression in tumors, in conjunction with clinicopathological features, could identify predictors of HCC recurrence and OS after curative hepatectomy. Our results indicated that the combination of high Axl and low LC3 expression significantly correlated with HCC recurrence and OS and that Axl and LC3 may serve as potential biomarkers for predicting HCC recurrence and OS.

## CONFLICT OF INTEREST

None declared.

## AUTHOR CONTRIBUTIONS

Hsu CC performed the experiments. Chen YS, Lo GH, Hsieh PM, Lin HY, and Chen YL collected the materials, reagents, and patient data. Dai CY, Huang JF, Chuang WL, and Yu ML analyzed the data. Lin CW designed the study and wrote the manuscript with Hsu CC. All of the authors made equally important recommendations for the manuscript and approved the final version of the manuscript.

## References

[1] Forner A , Llovet JM , Bruix J . Hepatocellular carcinoma. Lancet. 2012;379:1245‐1255. 22353262 10.1016/S0140-6736(11)61347-0 PMC13537732

[2] El‐Serag HB . Epidemiology of viral hepatitis and hepatocellular carcinoma. Gastroenterology. 2012;142(1264–1273):e1. 10.1053/j.gastro.2011.12.061 PMC3338949 22537432

[3] Lin CW , Lin CC , Mo LR , et al. Heavy alcohol consumption increases the incidence of hepatocellular carcinoma in hepatitis B virus‐related cirrhosis. J Hepatol. 2013;58:730‐735. 23220252 10.1016/j.jhep.2012.11.045

[4] Roayaie S , Obeidat K , Sposito C , et al. Resection of hepatocellular cancer </=2 cm: results from two western centers. Hepatology. 2013;57:1426‐1435. 22576353 10.1002/hep.25832 PMC3442120

[5] Forner A , Vilana R , Ayuso C , et al. Diagnosis of hepatic nodules 20 mm or smaller in cirrhosis: prospective validation of the noninvasive diagnostic criteria for hepatocellular carcinoma. Hepatology. 2008;47:97‐104. 18069697 10.1002/hep.21966

[6] Tabrizian P , Jibara G , Shrager B , Schwartz M , Roayaie S . Recurrence of hepatocellular cancer after resection: patterns, treatments, and prognosis. Ann Surg. 2015;261:947‐955. 25010665 10.1097/SLA.0000000000000710

[7] Lin CW , Chen YS , Lin CC , et al. Autophagy‐related gene LC3 expression in tumor and liver microenvironments significantly predicts recurrence of hepatocellular carcinoma after surgical resection. Clin Transl Gastroenterol. 2018;9:166. 29961754 10.1038/s41424-018-0033-4 PMC6026596

[8] Lin CW , Chen YS , Lin CC , et al. Significant predictors of OS in patients with hepatocellular carcinoma after surgical resection. PLoS ONE. 2018;13:e0202650. 30180193 10.1371/journal.pone.0202650 PMC6122804

[9] Toyoda H , Kumada T , Tada T , et al. Changes in highly sensitive alpha‐fetoprotein for the prediction of the outcome in patients with hepatocellular carcinoma after hepatectomy. Cancer Med. 2014;3:643‐651. 24591342 10.1002/cam4.218 PMC4101755

[10] Paccez JD , Vogelsang M , Parker MI , Zerbini LF . The receptor tyrosine kinase Axl in cancer: biological functions and therapeutic implications. Int J Cancer. 2014;134:1024‐1033. 23649974 10.1002/ijc.28246

[11] Koorstra J‐B , Karikari C , Feldmann G , et al. The Axl receptor tyrosine kinase confers an adverse prognostic influence in pancreatic cancer and represents a new therapeutic target. Cancer Biol Ther. 2009;8:618‐626. 19252414 10.4161/cbt.8.7.7923 PMC2678175

[12] Holland SJ , Pan A , Franci C , et al. R428, a selective small molecule inhibitor of Axl kinase, blocks tumor spread and prolongs survival in models of metastatic breast cancer. Cancer Res. 2010;70:1544‐1554. 20145120 10.1158/0008-5472.CAN-09-2997

[13] Vajkoczy P , Knyazev P , Kunkel A , et al. Dominant‐negative inhibition of the Axl receptor tyrosine kinase suppresses brain tumor cell growth and invasion and prolongs survival. Proc Natl Acad Sci USA. 2006;103:5799‐5804. 16585512 10.1073/pnas.0510923103 PMC1458653

[14] Reichl P , Fang M , Starlinger P , et al. Multicenter analysis of soluble Axl reveals diagnostic value for very early stage hepatocellular carcinoma. Int J Cancer. 2015;137:385‐394. 25529751 10.1002/ijc.29394 PMC4450342

[15] Wu X , Liu X , Koul S , Lee CY , Zhang Z , Halmos B . Axl kinase as a novel target for cancer therapy. Oncotarget. 2014;5:9546‐9563. 25337673 10.18632/oncotarget.2542 PMC4259419

[16] Xu J , Jia L , Ma H , Li Y , Ma Z , Zhao Y . Axl gene knockdown inhibits the metastasis properties of hepatocellular carcinoma via PI3K/Akt‐PAK1 signal pathway. Tumour Biol. 2014;35:3809‐3817. 24347489 10.1007/s13277-013-1521-5

[17] Reichl P , Dengler M , van Zijl F , et al. Axl activates autocrine transforming growth factor‐beta signaling in hepatocellular carcinoma. Hepatology. 2015;61:930‐941. 25251599 10.1002/hep.27492 PMC4450343

[18] Liu J , Wang K , Yan Z , et al. Axl expression stratifies patients with poor prognosis after hepatectomy for hepatocellular carcinoma. PLoS ONE. 2016;11:e0154767. 27182739 10.1371/journal.pone.0154767 PMC4868325

[19] Bruix J , Sherman M . Practice guidelines committee AAftSoLD. Management of hepatocellular carcinoma. Hepatology. 2005;42:1208‐1236. 16250051 10.1002/hep.20933

[20] Lin C‐W , Lin C‐C , Lee P‐H , et al. The autophagy marker LC3 strongly predicts immediate mortality after surgical resection for hepatocellular carcinoma. Oncotarget. 2017;8:91902‐91913. 29190884 10.18632/oncotarget.19763 PMC5696150

[21] Lee YJ , Hah YJ , Kang YN , et al. The autophagy‐related marker LC3 can predict prognosis in human hepatocellular carcinoma. PLoS ONE. 2013;8:e81540. 24282606 10.1371/journal.pone.0081540 PMC3839913

[22] Chen KD , Wang CC , Tsai MC , et al. Interconnections between autophagy and the coagulation cascade in hepatocellular carcinoma. Cell Death Dis. 2014;5:e1244. 24853422 10.1038/cddis.2014.212 PMC4047908

[23] Wu DH , Jia CC , Chen J , et al. Autophagic LC3B overexpression correlates with malignant progression and predicts a poor prognosis in hepatocellular carcinoma. Tumour Biol. 2014;35:12225‐12233. 25256671 10.1007/s13277-014-2531-7

